# Supplementary material for: Molecular Capture of Mycobacterium tuberculosis Genomes Directly from Clinical Samples: A Potential Backup Approach for Epidemiological and Drug Susceptibility Inferences
Source: Int J Mol Sci. 2023 Feb 2;24(3):2912. doi: 10.3390/ijms24032912 (PMC9918089; doi:10.3390/ijms24032912)
Supplement: Supplementary file 1 [file ijms-24-02912-s001.zip › Figures S1 and S2.pdf]

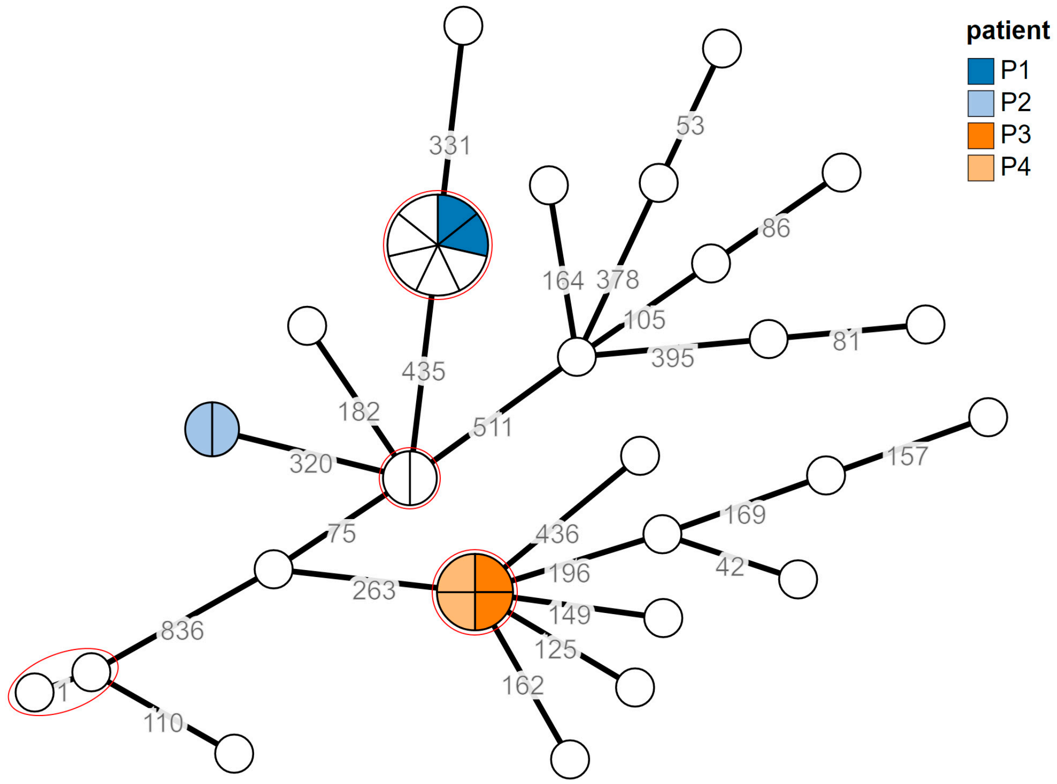

Figure S1. Core-genome phylogenetic analysis of 37 *M. tuberculosis* samples with (near) complete genome sequences. The minimum spanning tree was constructed based on a core-genome SNP-based alignment involving a total 3441 variant sites. Each circle (node) represents a unique SNP profile. Potential clusters (SNP distance below 12 SNPs) are highlighted by red colored circles. Data visualization was adapted from GrapeTree dashboard [see methods], with the colored nodes referring to the same-patient samples. Only samples yielding ≥90% horizontal coverage and at least 10-fold depth of coverage were included in the phylogenetic analysis.

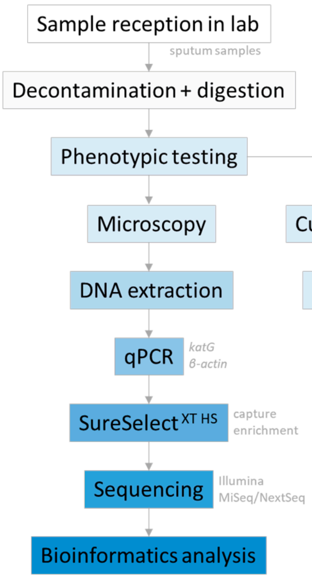

Figure S2. Procedure workflow for the capture and enrichment of *Mycobacterium tuberculosis* genomes directly from clinical samples. DST: drug susceptibility testing; RT-PCR: real time PCR.
